# Supplementary material for: Prediction of disability-free survival in healthy older people
Source: GeroScience. 2022 Apr 14;44(3):1641–55. doi: 10.1007/s11357-022-00547-x (PMC9213595; doi:10.1007/s11357-022-00547-x)
Supplement: Supplementary file 1 — (DOCX 418 kb) [file 11357_2022_547_MOESM1_ESM.docx]

**Supplementary Material to**

**“Prediction of disability-free survival in healthy older people”**

**Content**

[**1.** **Supplementary Methods** 2](#_Toc79607703)

[**2.** **Supplementary Figures** 5](#_Toc79607704)

[**3.** **Supplementary Tables** 9](#_Toc79607705)

# **Supplementary Methods**

Exclusion criteria

Exclusion criteria included a previous diagnosis of cardiovascular events (including myocardial infarction, heart failure, angina pectoris, stroke, transient ischemic attack, 50% carotid artery stenosis or previous carotid endarterectomy or stenting, coronary artery angioplasty or stenting, coronary artery bypass grafting, or abdominal aortic aneurysm), atrial fibrillation, evidence of dementia or major cognitive impairment, inability to perform independently any basic Katz activity of daily living (ADL), or a serious illness with a life expectancy of less than 5 years.

Definition of potential predictors collected at ASPREE baseline

1. Demographic variables
   - Age: At time of randomization
   - Sex: Male or female
   - Ethnicity/Race: White, Black, Hispanic, or others
   - Living status: Self-reported living at home alone or with family/friends/spouse
   - Years of education: Categorized as self-reported < 9 years, 9-11 years, 12 years, 13-15 years, 16 years, and 17-21 years.
2. Prevalent diseases or risk factors
   - Diabetes: Either self-reported, fasting glucose ≥ 126 mg/dL or intake of antidiabetic medication
   - Smoking history: Current or former/never smoker
   - Alcohol consumption: Current or former/never alcohol consumption
   - Family history of myocardial infarction: In a first degree relative at the age of < 50 years
3. Pathology
   - Non-high-density-lipoprotein-cholesterol: Measured in fasting blood sample
   - High-density-lipoprotein-cholesterol: Measured in fasting blood sample
   - eGFR: Estimated using the CKD formula
   - Haemoglobin: Measured in fasting blood sample
4. Body readings
   - Systolic blood pressure: Mean of three consecutive measurements in a resting position
   - Diastolic blood pressure: Mean of three consecutive measurements in a resting position
   - Body-mass-index: Body weight (kg) / Height (m)^2^
   - Abdominal circumference: cm
   - Gait speed: m/s
   - Grip strength: Assessed on the dominant hand in three consecutive measurements using a dynamometer. For the analyses the mean value was used.
5. Medication
   - Antihypertensive agents: Defined by the intake of agents from the ATC code categories C02, C03, C07, C08, and C09.
   - Lipid-lowering agents: Defined by the intake of agents from the ATC code category C10AA.
   - Randomization to aspirin treatment: As per randomization of the ASPREE trial.
6. Assessment of cognitive function and depression
   - 3MS score: The Modified Mini-Mental State (3MS) examination was assessed using a standardized questionnaire scored out of 100 points.
   - CES-D-10 score: Centre for Epidemiological Studies – Depression 10 question instrument. For the present analyses a binary cut-off of ≥ 8 was used.

Statistical analyses

*Prognostic model development*

The combination with bootstrap can be described as follows: for each imputed data set, we randomly drew 100 bootstrap samples with replacement, and performed group-lasso selection on each bootstrap sample. The variable inclusion frequency (VIF) over 100*5 = 500 models was calculated for each predictor. A variable with VIF ≥ 60% was then included in the final model. The final model was derived by refitting the selected predictors to each of the 5 imputed datasets, and combining parameter estimates using Rubin’s rule.^34^ Variable selection based on VIF has previously demonstrated good performance in the presence of imputed data.^35^

Rubin’s rule: suppose $\hat{\theta_{i}}$ is a regression coefficient obtained from $i^{th}$ imputed data set and $W_{i}$ is the estimated variance of $\hat{\theta_{i}}$. the combined estimate $\hat{\theta}$ is the average of the individual estimates

$$\hat{\theta}=\frac{1}{m}\sum_{i=1}^{m} \hat{\theta_{i}}$$

With *m* is the number of imputation data sets.

The total variance of $\hat{\theta}$ is formed from the within imputation variance $W=(\frac{1}{m})\sum_{i=1}^{m} W_{i}$ and the between imputation variance $B=(\frac{1}{m-1})\sum_{i=1}^{m} \left( \hat{\theta_{i}}-\hat{\theta} \right)^{2}$

$$var\left( \hat{\theta} \right)=W+\left( 1+\frac{1}{m} \right)B$$

*Model performance*

The area under the cumulative/dynamic ROC curve (AUC) at 5 years was used to assess discrimination.^36^ AUC ranges from 0.5 to 1 with a higher value indicating better ability to discriminate those who developed events and those who did not. Harrell’s calibration plot at 5 years was used to assess the agreement between predicted and observed risks.^37^ The apparent performances were obtained by evaluating the final model on the development samples (averaged across 5 imputed data sets).

Harrel’s calibration plot: Participants are first stratified into risk groups (normally 5-10) based on quantile of their predicted risks at a given time point. Next, the average predicted risks for each stratum are plotted against the respective average observed risk (based on Kaplan-Meier estimates)

*Model validation*

To quantify the degree of optimism due to overfitting in performance measures, we implemented internal validation using the enhanced bootstrap resampling procedure.^37,38^ The optimism was calculated as follows. From the original data, 100 random bootstrap samples were drawn. For each sample, we repeated the model development procedure as outlined above to obtain a bootstrap final model. We then calculated the difference between bootstrap apparent performance (averaged across bootstrap imputed data) and bootstrap test performance (averaged across original imputed data). Finally, these differences were averaged across 100 bootstrap samples to obtain the single estimate for the optimism. The procedure is illustrated in Figure S2. The estimated optimism was then subtracted from the apparent performance to obtain the bias-corrected predictive performance.

# **Supplementary Figures**

Figure S1: Overview of model building and validation process.


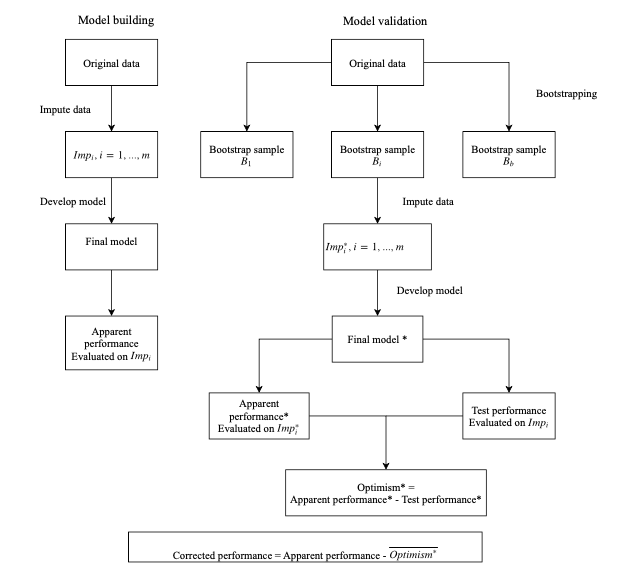


Figure S2: Visualizing the non-linear effects of eGFR and BMI in the model for males.


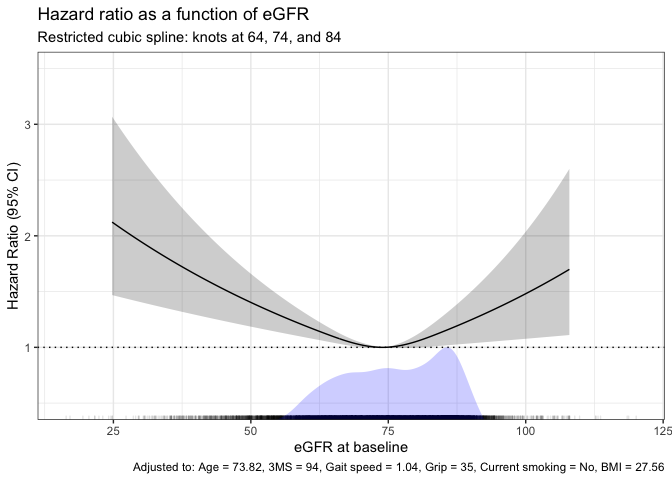


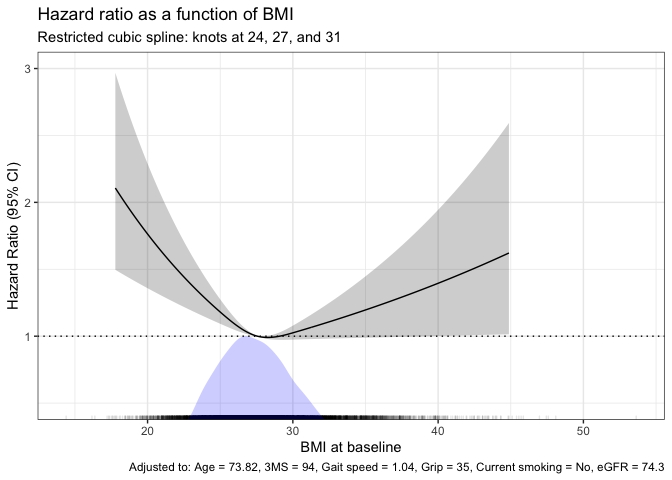


Reference level HR = 1: (left) eGFR = 74.30 (mL/min per 1.73 m^2^); (right) BMI = 27.56 (kg/m^2^). The grey shaded area indicates the 95% confidence interval. Density functions (blue shaded area) of eGFR and BMI were superimposed.

Figure S3: Visualizing the non-linear effects (black lines) of gait speed and BMI in the model for females.


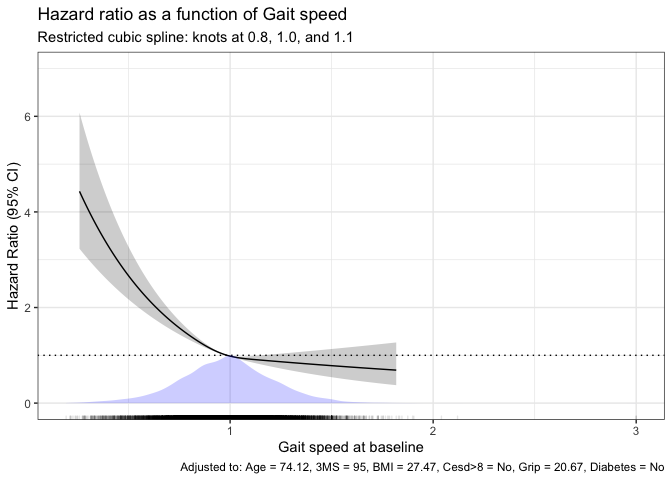


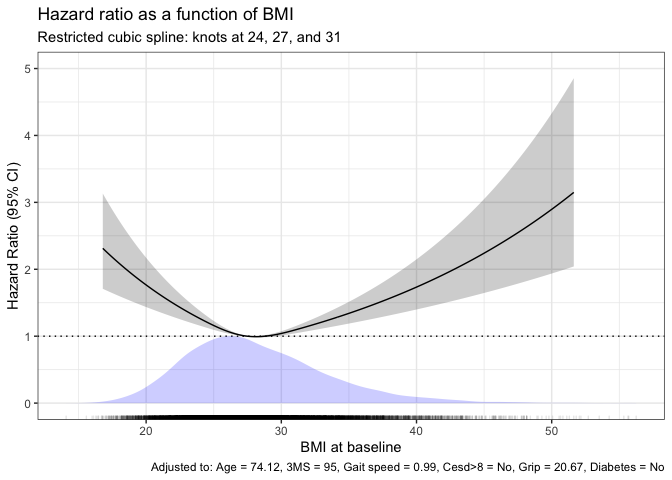


Reference level HR = 1: (left) Gait speed = 0.98 (m/s); (right) BMI = 27.47 (kg/m^2^). The grey shaded area indicates the 95% confidence interval. Density functions (blue shaded area) of Gait speed and BMI were superimposed.

Figure S4: Risk distribution for each age group in males and females.

| Female | 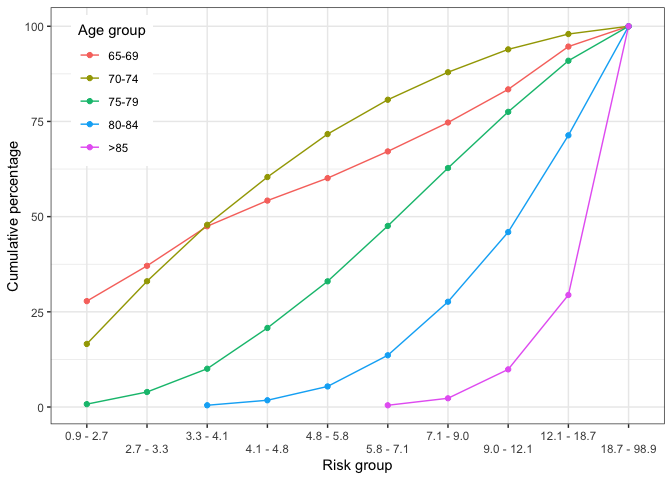 |
| --- | --- |
| Male | 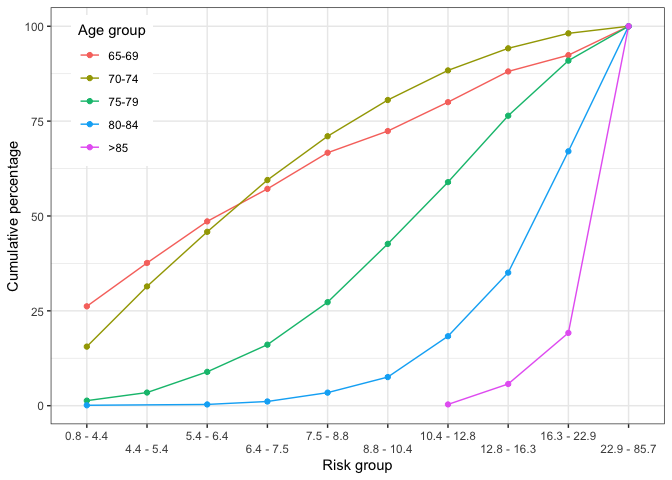 |

# **Supplementary Tables**

Table S1: Number of missing values in a case

| All participants | | | Female | | Male | |
| --- | --- | --- | --- | --- | --- | --- |
| N missing in a case | N | % | N | % | N | % |
| 0 | 16,327 | 85.42 | 9,180 | 85.14 | 7,147 | 85.78 |
| 1 | 2,197 | 11.49 | 1,276 | 11.83 | 921 | 11.05 |
| 2 | 431 | 2.25 | 234 | 2.17 | 197 | 2.36 |
| 3 | 141 | 0.74 | 83 | 0.77 | 58 | 0.70 |
| 4 | 18 | 0.09 | 9 | 0.08 | 9 | 0.11 |

This table shows the number of missing variables for each participant. This means, that e.g. 16,327 participants did not have any missing values, while 2,197 had one missing value. Abbreviations: N = Number.

Table S2: Break-down of endpoint events during 5-year follow-up.

|  | Female | Male |
| --- | --- | --- |
| Total number of events | N = 795 | N = 799 |
| - - - Death | 344 (43%) | 428 (54%) |
| - - - Dementia | 251 (32%) | 229 (29%) |
| - - - Persistent Physical Disability | 200 (25%) | 142 (18%) |

Table S3: Variable selection frequency based on the least absolute shrinkage and selection operator. Variables in grey were selected for the final models.

| Variable | Male | Female |
| --- | --- | --- |
| Age | 100.0 | 100.0 |
| 3MS | 100.0 | 100.0 |
| Gait speed | 100.0 | 100.0 |
| BMI | 86.8 | 98.6 |
| CES-D | 8.2 | 97.0 |
| Grip strength | 98.8 | 92.8 |
| Diabetes | 26.2 | 65.8 |
| Ethnicity/Race | 6.6 | 39.6 |
| HDL-c | 27.6 | 38.4 |
| Smoking | 99.8 | 36.6 |
| Alcohol consumption | 26.6 | 36.2 |
| Living condition | 24.0 | 31.8 |
| Waist circumference | 5.8 | 26.8 |
| eGFR | 76.4 | 22.2 |
| Family history of MI | 6.0 | 17.0 |
| Antihypertensive agents | 0.4 | 15.6 |
| Haemoglobin | 52.8 | 8.4 |
| Education | 3.0 | 7.2 |
| Non-HDL-c | 50.2 | 4.6 |
| DBP | 2.8 | 4.6 |
| Aspirin treatment | 5.4 | 4.2 |
| SBP | 6.4 | 3.0 |
| Lipid-lowering agents | 9.8 | 2.0 |

Abbreviations: 3MS = Modified Mini-Mental State examination, HDL-c = high-density-lipoprotein-cholesterol, CES-D = Centre for Epidemiologic Studies—Depression 10 question assessment, MI = myocardial infarction, eGFR = estimated glomerular filtration rate, DBP = diastolic blood pressure, SBP = systolic blood pressure.

Table S4: AUC at 5 years of each predictor, of their combination when added sequentially in order of their AUC, and of the final models.

| **Variable** | **AUC** | **Combination** | **AUC** |
| --- | --- | --- | --- |
| **Male** | | | |
| Age | 0.65 | Age | 0.65 |
| 3MS | 0.64 | Age + 3MS | 0.69 |
| Gait speed | 0.62 | Age + 3MS + Gait speed | 0.71 |
| Smoking | 0.52 | Age + 3MS + Gait speed + Smoking | 0.72 |
| Grip strength | 0.62 | Age + 3MS + Gait speed + Smoking + Grip strength | 0.72 |
| BMI | 0.54 | Age + 3MS + Gait speed + Smoking + Grip strength + BMI | 0.73 |
| eGFR | 0.55 | **Final model: Age + 3MS + Gait speed + Smoking + Grip strength + BMI + eGFR** | 0.73 |
|  |  | **Final model (bias-corrected)** | **0.72** |
| **Female** | | | |
| Age | 0.66 | Age | 0.66 |
| 3MS | 0.67 | Age + 3MS | 0.71 |
| Gait speed | 0.68 | Age + 3MS + Gait speed | 0.75 |
| BMI | 0.56 | Age + 3MS + Gait speed + BMI | 0.76 |
| CES-D | 0.53 | Age + 3MS + Gait speed + BMI + CES-D | 0.76 |
| Grip strength | 0.60 | Age + 3MS + Gait speed + BMI + CES-D + Grip strength | 0.76 |
| Diabetes | 0.53 | **Final model: Age + 3MS + Gait speed + BMI + CES-D + Grip strength + Diabetes** | 0.76 |
|  |  | **Final model (bias-corrected)** | **0.75** |
|  | | | |

Abbreviations: AUC = area under the curve, 3MS = Modified Mini-Mental State examination, CES-D = Centre for Epidemiologic Studies—Depression 10 question assessment, BMI = body mass index, eGFR = estimated glomerular filtration rate.

Table S5: Illustrations of the risk computational formula.

|  | **Female** | **Male** |
| --- | --- | --- |
| Age | 75 | 75 |
| 3MS | 95 | 95 |
| Gait speed | 0.5 | 0.7 |
| Grip strength | 15 | 18 |
| BMI | 35 | 40 |
| Smoking | - | Current |
| CES-D ≥ 8 | Yes | - |
| Diabetes | No | - |
| eGFR | - | 45 |
| **Linear Predictor** | 0.0812863 x 75 - 0.0785434 x 95 -2.1058784 x 0.5 - 0.0824954 x 35 + (35 - 24.48889)^3 x 0.0022333 -  (35 - 27.46667)^3 x 0.0040281 +  (35 - 31.17188)^3 x 0.0017948 + 0.3885922 - 0.0188137 x 15 = -4.2269 | 0.0791378 x 75 - 0.0614952 x 95 -0.9872901 x 0.7 - 0.0175855 x 18 -  0.0809386 x 40 +  (40 - 25.29407)^3 x 0.0034296 -  (40 - 27.55675)^3 x 0.0064518 +  (40 - 30.12438)^3 x 0.0030222 -0.0157298 x 45 = -3.4719 |
| **Estimated 5- year risk** | **(1 – (4.787198 x 10^-8^ )^exp (-4.2269)^) x 100% = 21.81%** | **(1 – (5.304956 x 10^-8^)^exp (-3.4719)^) x 100% = 40.56%** |
