# Supplementary material for: Prediction of disability-free survival in healthy older people
Source: GeroScience. 2022 Apr 14;44(3):1641–55. doi: 10.1007/s11357-022-00547-x (PMC9213595; doi:10.1007/s11357-022-00547-x)
Supplement: Supplementary file 2 — (DOCX 309 kb) [file 11357_2022_547_MOESM2_ESM.docx]

**Prediction of disability-free survival in healthy older people**

Running title: Prediction of disability-free survivaL

Johannes Tobias Neumann, MD, MCR^1,2,3,*^ ; Le T. P. Thao, PhD^1,*^; Anne M. Murray, MD, MSc^4,*^; Emily Callander, PhD^1^; Prudence R. Carr, PhD^1^; Mark R. Nelson, PhD^1,5^; Rory Wolfe, PhD^1^; Robyn L. Woods, PhD^1^; Christopher M. Reid, PhD^1,6^; Raj C. Shah, MD^7^; Anne B. Newman, MD, MPH^8^; Jeff D. Williamson, MD, MHS^9^; Andrew M. Tonkin, MD^1^; John J. McNeil, PhD^1^, on behalf of the ASPREE investigators.

**Affiliations:**

1. Department of Epidemiology and Preventive Medicine, School of Public Health and Preventive Medicine, Monash University, Melbourne, Australia.
2. Department of Cardiology, University Heart & Vascular Centre, Hamburg, Hamburg, Germany.
3. German Centre for Cardiovascular Research (DZHK), Partner Site Hamburg/Kiel/Lübeck, Hamburg, Germany.
4. Division of Geriatrics, Department of Medicine, Hennepin Healthcare, and Berman Centre for Outcomes and Clinical Research, Hennepin Healthcare Research Institute, Minneapolis, United States of America.
5. Menzies Institute for Medical Research, University of Tasmania, Hobart, Australia.
6. Curtin School of Population Health, Curtin University, Perth, WA, Australia.
7. Department of Family Medicine and Rush Alzheimer's Disease Centre, Rush University Medical Centre, Chicago, Illinois, United States of America.
8. Centre for Aging and Population Health, Department of Epidemiology, University of Pittsburgh, Pittsburgh, United States of America.
9. Sticht Centre on Health Aging and Alzheimer’s Prevention, Section on Gerontology and Geriatric Medicine, Department of Internal Medicine, Wake Forest School of Medicine, Winston-Salem, NC, United States of America.

* These authors contributed equally and should be considered as shared first authors.

**Word count:** 3,317/3,500 words

**Address for correspondence:**

Johannes T. Neumann

Department of Epidemiology and Preventive Medicine

School of Public Health and Preventive Medicine

99 Commercial Road

3004 Melbourne, Victoria, Australia

Email: Johannes.neumann@monash.edu

**Abstract (246 words)**

Background: Prolonging survival in good health is a fundamental societal goal. However, the leading determinants of disability-free survival in healthy older people have not been well established.

Methods: Data from ASPREE, a bi-national placebo-controlled trial of aspirin with 4.7 years median follow-up, was analysed. At enrolment, participants were healthy and without prior cardiovascular events, dementia, or persistent physical disability. Disability-free survival outcome was defined as absence of dementia, persistent disability, or death. Selection of potential predictors from amongst 25 biomedical, psychosocial and lifestyle variables including recognized geriatric risk factors, utilizing a machine-learning approach. Separate models were developed for men and women. The selected predictors were evaluated in a multivariable Cox proportional hazards model and validated internally by bootstrapping.

Results: We included 19,114 Australian and US participants aged ≥65 years (median 74 years, IQR 71.6-77.7). Common predictors of a worse prognosis in both sexes included higher age, lower Modified Mini-Mental State Examination score, lower gait speed, lower grip strength, and abnormal (low or elevated) body mass index. Additional risk factors for men included current smoking, and abnormal eGFR. In women, diabetes and depression were additional predictors. The biased-corrected areas under the receiver operating characteristic curves for the final prognostic models at 5 years were 0.72 for men and 0.75 for women. Final models showed good calibration between the observed and predicted risks.

Conclusion: We developed a prediction model in which age, cognitive function and gait speed were the strongest predictors of disability-free survival in healthy older people.

Trial registration: Clinicaltrials.gov (NCT01038583)

Keywords: Risk prediction, disability, survival, elderly, healthy, public health.

**Introduction**

Currently, approximately 900 million individuals worldwide are aged 60 years or older and this number is expected to nearly double by 2050.(1) Amongst those reaching this age in good health, future years of life will commonly be marked by functional decline involving physical disability or cognitive impairment, leading to a loss of independence and significant costs to society.(2)

The ability to prolong the time spent in good health, living without dependence on others, has become a fundamental societal goal.(3-5) For this reason disability-free survival has emerged as an important geriatric research outcome in contrast to disease-specific outcomes such as cardiovascular disease or dementia.^4^ Particularly in older people, the maintenance of good health requires the avoidance of multiple interacting co-morbidities and chronic conditions, many with shared risk factors and management strategies.(6-8) Understanding the major preventable determinants, not only of individual diseases, but ongoing good health(9) can be facilitated by development of prediction models for disability-free survival.

Previous studies have identified risk factors for a range of specific geriatric outcomes including frailty, physical disability, dementia, and death. In addition to age, these have included abnormal body-mass-index (BMI), smoking, diabetes, abnormal blood lipids, chronic kidney disease, low level of physical activity, low gait speed, presence of depression, subclinical cardiovascular disease, specific diets, and lack of social support.(10-19) These studies have typically been limited to the evaluation of pre-specified relationships between predictors and specific outcomes, rather than a composite measure of functional independence. Similarly, no previous study has employed a machine-learning approach to enable objective variable selection.(20)

The ASPirin in Reducing Events in the Elderly (ASPREE) placebo-controlled trial of low-dose aspirin was the first large-scale clinical trial that utilized disability-free survival as the prespecified primary endpoint. Measures of physical disability and cognition were assessed systematically throughout follow-up in all subjects in detail, which is rarely available in a non-trial setting. In this analysis, we used the ASPREE data to develop and validate a prediction model for disability-free survival in a population of relatively healthy individuals aged 65 or more when recruited. Machine-learning techniques allowed the relative importance of key predictors to be determined in an unbiased and objective fashion. Specifically, we sought to identify modifiable risk factors that could identify individuals at risk and prioritize preventive policies.

**Methods**

Study population and trial design

ASPREE was a large, randomized, double-blind, placebo-controlled trial investigating the efficacy of 100mg aspirin on extending disability-free survival in a healthy elderly population. The trial design and primary results have been published previously.(21-24) Briefly, 19,114 community-dwelling individuals without prior cardiovascular events, dementia or physical disability were randomized to low-dose enteric coated aspirin or placebo and followed for an average of 4.6 years. All individuals were at least 70 years old (≥ 65 years for US minorities). Exclusion criteria included a previous diagnosis of cardiovascular events (including myocardial infarction, heart failure, angina pectoris, stroke, transient ischemic attack, 50% carotid artery stenosis or previous carotid endarterectomy or stenting, coronary artery angioplasty or stenting, coronary artery bypass grafting, or abdominal aortic aneurysm), atrial fibrillation, evidence of dementia or major cognitive impairment, inability to perform independently any basic Katz activity of daily living (ADL), or a serious illness with a life expectancy of less than 5 years. All participants provided written informed consent. The trial was approved by the local Ethics Committees and is registered on clinicaltrials.gov (NCT01038583). Requests for data access will be via the ASPREE Principal Investigators with details for applications provided through the web site, www.ASPREE.org

Baseline collection, definition, and selection of potential predictors

A total of 25 candidate predictors were selected on the basis of previous outcome studies related to dementia, disability and death(11, 17-19, 25), and their likely availability in typical clinical practice. The preselection of candidate predictors was also performed to minimize the possibility of including noise variables in the final model. The variables were collected as part of the initial standardized screening process prior to participant enrolment in ASPREE and included:

- demographics (age, living status, years of education, ethnicity/race),
- prevalent conditions or risk factors (diabetes, smoking history, alcohol consumption, family history of myocardial infarction),
- laboratory results (high-density-lipoprotein-cholesterol [HDL-c], non-HDL-c, estimated glomerular filtration rate [eGFR], haemoglobin),
- physical measurements (systolic and diastolic blood pressure, BMI, abdominal circumference, grip strength, gait speed),
- medication use (antihypertensive agents, lipid-lowering agents, and in-trial randomization to aspirin treatment),
- measures of cognitive function (Modified Mini-Mental State Examination [3MS] score) and depression (Centre for Epidemiological Studies-Depression-10 questions [CES-D] score, as a binary variable ≥ 8).

Details related to all predictors are provided in the *Supplementary Material*.

Outcome ascertainment

The primary end point of disability-free survival was defined as survival free from dementia or persistent physical disability, and was used as a surrogate for functional independence.(21) Physical disability and dementia were combined in the primary end point, as they represent the important reasons why individuals lose the ability to live independently. Participants without the documented outcome were censored at 5 years or at the last date they were known to be event-free, whichever came first. To assess this composite endpoint, all participants had serial in-person visits, which included standardized assessments of cognitive function with a cognitive battery, and dementia assessment if triggered, and physical disability, defined as an inability to perform, having severe difficulty in performing, or requiring assistance to complete at least one of the six basic ADL by self-report.(26) This was considered to be persistent if the same ADL loss was confirmed six months later.

Dementia was adjudicated based on the Diagnostic and Statistical Manual of Mental Disorders IV (DSM-IV) criteria.(27) The ascertainment of death has been described before and was based on information collected from at least two sources including close contacts, physicians, public death notices and by linkage to the Australian National Death Index and US National Death Index.(23) Dementia and cause of death were adjudicated and confirmed by the respective endpoint committees blinded to treatment allocation.

Statistical analyses

*Prognostic model development*

Separate models were developed for men and women due to their known differing risk factor profiles. The statistical model employed was the Cox proportional hazards regression model. Prior to selection of variables, we tested for non-linearity of continuous variables. Specifically, a complete case analysis was initially fitted with all candidate predictors, in which each continuous variable was modelled with a restricted cubic spline function with 2 degrees of freedom. Statistically significant non-linear relations were found for BMI, HDL-c, waist circumference, and gait speed in the model for women; and non-HDL-c, eGFR, BMI and waist circumference in the model for men.

Data was missing for 11 variables with the proportion ranging from very low, <0.1% (for years of education, haemoglobin, and CES-D) to as high as 8% (for family history of myocardial infarction). The total proportion of missing values was 1% but using a complete case analysis would have discarded about 15% of the sample size (see Table S1). Hence, multiple imputation was performed using chain equations, assuming data were missing at random.(28) The imputation model included all candidate predictors and other potentially relevant variables. Non-linear BMI, waist circumference, gait speed, eGFR, and non-HDL-c were imputed passively.(29) The survival outcomes were included as a combination of Nelson-Aalen estimator of the cumulative hazard function and the outcome status.(30) Five data sets were imputed for men and women separately, based on the amount of missing data.(31)

Variable selection was performed using the machine-learning technique “group least absolute shrinkage and selection operator” (group-lasso) in combination with bootstrapping.(32) The group-lasso model ensures that categorical variables, or linear and non-linear terms of continuous variables are included or excluded in the model all together. The penalty parameter, which regulates the amount of shrinkage, was selected by optimizing the 10-fold cross-validation prediction error. We chose a suboptimal penalty based on the “one-standard-error” rule to obtain a more parsimonious model.(33)

The combination with bootstrap can be described as follows: for each imputed data set, we randomly drew 100 bootstrap samples with replacement, and performed group-lasso selection on each bootstrap sample. The variable inclusion frequency (VIF) over 100*5 = 500 models was calculated for each predictor. A variable with VIF ≥ 60% was then included in the final model. The final model was derived by refitting the selected predictors to each of the 5 imputed datasets, and combining parameter estimates using Rubin’s rule.(34) Variable selection based on VIF has previously demonstrated good performance in the presence of imputed data.(35)

*Model performance*

The area under the cumulative/dynamic ROC curve (AUC) at 5 years was used to assess discrimination.(36) AUC ranges from 0.5 to 1 with a higher value indicating better ability to discriminate those who developed events and those who did not. Harrell’s calibration plot at 5 years was used to assess the agreement between predicted and observed risks.(37) The apparent performances were obtained by evaluating the final model on the development samples, which were used to build the models (averaged across 5 imputed data sets).

*Model validation*

To quantify the degree of optimism due to overfitting in performance measures, we implemented internal validation using the enhanced bootstrap resampling procedure.(37, 38) The optimism was calculated as follows. From the original data, 100 random bootstrap samples were drawn. For each sample, we repeated the model development procedure (including imputation and lasso selection) as outlined above to obtain a bootstrap final model. We then calculated the difference between bootstrap apparent performance (averaged across bootstrap imputed data) and bootstrap test performance (averaged across original imputed data). Finally, these differences were averaged across 100 bootstrap samples to obtain the single estimate for the optimism. The procedure is illustrated in Figure S1. The estimated optimism was then subtracted from the apparent performance to obtain the bias-corrected predictive performance.

All analyses were conducted using R version 4.0.2 with the companion R packages: mice, grpreg, rms, survival, timeROC, pec.

**Results**

Population characteristics

A total of 19,114 community-dwelling Australian and US ASPREE participants were included in this study. The median age of the trial population was 74 years (interquartile range [IQR] 71.6-77.7) and 10,782 (56.4%) were women. Baseline characteristics for the overall population of men and women are reported in Table 1.

During the 5-year follow-up (median (IQR) 4.6 (3.5-5.6) years), 795 (7.3%) female participants and 799 (9.6%) male participants developed dementia or persistent physical disability or died. Death accounted for 54% of the total events in men and 43% in women (Table S2).

Model development for risk of the primary composite outcome

Results of the variable inclusion frequency are shown in Table S3. The multivariable Cox regression models which included variables with VIF ≥ 60% are presented in Table 2. Specifically, increasing age, lower 3MS score, lower gait speed, lower grip strength, and abnormal (low or elevated) BMI were identified as risk factors in both sexes. In men, current smoking and eGFR were additionally selected as predictors. In women, the presence of diabetes and a CES-D score ≥ 8 were also selected as predictors.

The final models (Table 2) show that a 5-year increase in age corresponded to 49% and 50% increased risk of not maintaining disability-free survival in men and women respectively, while a 5-unit increase in 3MS corresponded to about 30% decreased risk in both sexes. A decrease in gait speed from 1.0 to 0.5 m/s corresponded to 64% increased risk in men, and nearly 3-fold higher risk in women. Figure S3 illustrates the non-linear relation between gait speed and the primary outcome for women. Importantly, gait speed less than 1 m/s was associated with a substantially greater negative impact on risk of disability-free survival relative to the lesser beneficial impact of faster gait speed. The relationship between BMI and the outcome was non-linear in both models (Figure S2, S3). The risk was minimal with a BMI of around 27 kg/m^2^, whereas both elevated and lower BMI values were associated with an unfavourable outcome.

In women, having diabetes and depression (CES-D ≥ 8) were associated with 30% and 47% increased risk of the primary composite outcome, respectively. In men, current smokers had almost 2-fold higher risk of earlier onset of death, dementia or disability compared to former/never smokers. The non-linear relation of eGFR (Figure S2) indicated that compared to a reference value of 74, a higher or lower value of eGFR was associated with a higher risk.

Model performance and calibration

The discriminative performance of each predictor and their combinations for the prediction of disability-free survival are presented in Figure 1 and Table S4. The AUCs of age alone were 0.66 and 0.65 in women and men, respectively. Adding 3MS increased the discriminative performance by about 23% in both sexes. Compared to the simplified model which consisted of age and 3MS, the final models improved the predictive performance by 24% (AUC 0.76 vs. 0.71) in women and 21% (AUC 0.73 vs. 0.69) in men (Table S4). After correcting for optimism, the final models showed good discrimination, with an AUC of 0.72 for males and 0.75 for women (Table S4).

The final models also showed good agreement between the observed and the predicted risk of the overall outcome, although the risk for men was slightly overestimated in the higher risk categories (Figure 2). The predicted probability of the endpoint at 5 years can be calculated using the formula provided in Table 3. The illustrations for two specific participants are displayed in the supplementary material (Table S5).

The risk distribution within each 5-year increment age group was further investigated in Figure S4. As expected, the figure demonstrates the strong association between advanced age and event risk in both sexes. Compared to older age groups (75-85+), younger age groups (65-74) appear to have a lower proportion of people in the lower risk categories.

**Discussion**

We utilized a machine-learning variable selection approach to identify the determinants of survival free of persistent physical disability and dementia in the ASPREE study population.(21) Disability-free survival is a surrogate measure of functional independence and was assessed by regular in-person surveillance of a group of 19,114 apparently healthy individuals participating in a large-scale trial of low-dose aspirin. All participants were free of prior cardiovascular events, disability, and dementia at the commencement of the study.

During a median follow-up of 4.7 years the average age increased from 74.0 to 78.8 years and 1,594 individuals became disabled or died. By choosing a population relatively healthy at baseline and regularly monitoring each individual for the onset of dementia or physical disability, this study provided a unique opportunity to identify and prioritize the characteristics of those most likely and those least likely to continue living independently with increasing age. A particular strength of these analyses was the objective approach used for variable selection, which was based on a machine-learning technique applied to a range of clinical variables measured at study entry, together with previously recognized risk factors for physical and cognitive decline.

Out of 25 potential biomedical and social predictors tested, 7 contributed substantially to the prediction of disability-free survival in women and 7 were predictors in men. As expected, advanced age was the most important predictor, which alone resulted in an AUC of 0.65. Cognitive function was the second most important contributor followed by gait speed. Importantly, these three predictors alone resulted in the highest AUC increase, which empathizes their importance. Additional predictors in men included grip strength, smoking, BMI, and renal function. In women, diabetes, and depression scores also contributed to the model. In the case of eGFR and BMI, the association were non-linear and U-shaped, similar to that noted in prior studies of mortality and other geriatric outcomes.

Previous studies have identified similar risk factor profiles as predictors of cognitive decline, frailty, functional decline, and a range of other chronic diseases of ageing.(10-19) For example, a high BMI, slow gait speed and/or the presence of diabetes or hypertension have been identified as predictors of cognitive decline. Similarly slowing gait speed, weaker handgrip, increased BMI, and diabetes have been identified as predictors of physical decline and frailty. The interdependence of several of these predictors creates a web of causation in which physical disability, dementia or death can be the end result of several pathways.

The dominant impact of cognitive decline and reduced physical function suggests the possibility that interventions designed to improve cognition or physical function might alter the trajectory of decline in otherwise healthy older subjects. Recently published trials suggested that modest improvement may be possible. Amongst these: first, the LIFE study showed that the initiation of a moderate intensity physical program in sedentary individuals aged 70-89 years modestly reduced progression to major mobility disorder, (assessed as the ability to walk 400metres). The program involving aerobic, resistance and flexibility training provided in centre twice weekly and twice weekly at home.(39) The intervention over 2.6 years did not affect cognitive decline or reduce the incidence of cardiovascular disease.(40) Second, the SPRINT-MIND trial showed a reduction in the combined outcome of mild cognitive impairment plus probable dementia amongst older individuals on intensified blood pressure management.(41) The study involved 9,361 hypertensive older adults with increased cardiovascular risk whose mean systolic BP was 121.4 mmHg in the intensive-treatment group and 136.2 mmHg in the standard group. Third, the Finnish Geriatric Intervention Study to Prevent Cognitive Impairment and Disability (FINGER) trial investigated the efficacy of a multidomain lifestyle intervention including dietary guidance, physical activity, cognitive training, and monitoring and management of cardiovascular risk factors. The control group received general health advice. After 2 years, the intervention group improved more in executive function, processing speed, and complex memory tasks regardless of genetic risk or baseline risk factor levels.

From a clinical perspective, the identification of cognitive decline and physical limitations indicate that an individual is tracking towards an earlier loss of independence. The results of the trials described above suggest that the loss of independence in older individuals may be slowed by a multifactor intervention. However, the likelihood of these interventions producing a significant slowing of the trajectory towards loss of independence is as yet unclear.

From a population perspective, the results also emphasize the relevance of addressing the determinants of physical and cognitive decline at an earlier stage in the life-course. Although diet and lipids were not included as predictive factors in the final model, their role as shared determinants in the development of cognition and physical capacity at earlier stages has been well established, and they should be included in any strategy to promote healthy ageing. Furthermore, BMI and diabetes were part of the final models. A 2015 forecast of future trends in disability and life expectancy in the UK predicted an increase of 25% (over 10 years) in the number with care needs, with one quarter of the remaining life expectancy of those aged 65 years or above expected to involve a significant disability.(42) Although the increase in prevalence is largely a reflection of population ageing, it emphasizes the importance of strategies to extend disability-free survival. However, as our study results were based on an older population, future longitudinal studies are warranted to evaluate the impact of these determinants.

Strengths of this study include the extensive data collected from a large population with relatively small amounts of missing data. The measurement of disability-free survival required data concerning the time of onset of physical disability, dementia, and death, with diagnoses of dementia confirmed by a specialist adjudication panel; systematic collection of information of this type is rarely available outside the context of a clinical trial. The outcome data was accompanied by measurement of a wide range of clinically relevant and recognized geriatric potential prediction variables (biomedical and social) collected near the time of study initiation.

The limitations include that the majority of the study population were white Caucasians, most of whom lived in Australia, a high-income country with universal healthcare services. As a whole, the cohort had extensive use of preventive medications including statins (in 30%) and anti-hypertensive agents (in 35%), indicating a high level of preventive care. The generalizability of the study is also restricted to individuals reaching the 70-plus age group in relatively good health free of prior cardiovascular disease, dementia, or significant physical limitations. It is also possible, that there is residual confounding, and future research may identify new, relevant predictors. Finally, we were not able to perform an external validation, as comparable datasets are missing.

In conclusion, in a population of healthy older people, both modifiable and non-modifiable characteristics successfully identified individuals at higher risk of dying or developing dementia or physical disability within 5 years. After age, a low 3MS score was the strongest predictor followed by slow gait speed with a lesser contribution by other risk factors. Recent trials suggest that interventions aimed at slowing cognitive and physical decline are effective but additional trials will be required to demonstrate a meaningful impact in prolonging the time individuals remain functionally independent.

**Ethical statement:** The trial was approved by the local Ethics Committees and is registered on clinicaltrials.gov (NCT01038583). All participants provided written informed consent.

**Disclosures:** No conflicts were reported.

**Acknowledgements:**We thank the ASPREE trial staff, participants, and general practitioners.

**Funding:**Supported by the National Institute on Aging and the National Cancer Institute at the National Institutes of Health (grant numbers U01AG029824, U19AG062682); the National Health and Medical Research Council of Australia (grant numbers 334047, 1127060); Monash University and the Victorian Cancer Agency. J.N. is recipient of a fellowship by the Deutsche Forschungsgemeinschaft (NE 2165/1-1). C.M.R. is supported through a NHMRC Principal Research Fellowship (APP 1136372). JMCN is supported through an NHMRC Leadership Fellowship (IG 1173690).

**References**

1. World Health Organization. World report on ageing and health. 2015.

2. Disease GBD, Injury I, Prevalence C. Global, regional, and national incidence, prevalence, and years lived with disability for 354 diseases and injuries for 195 countries and territories, 1990-2017: a systematic analysis for the Global Burden of Disease Study 2017. Lancet. 2018;392(10159):1789-858.

3. Fried LP, Rowe JW. Health in Aging - Past, Present, and Future. N Engl J Med. 2020;383(14):1293-6.

4. Rowe JW, Kahn RL. Successful aging. Gerontologist. 1997;37(4):433-40.

5. Jacob ME, Yee LM, Diehr PH, Arnold AM, Thielke SM, Chaves PH, et al. Can a Healthy Lifestyle Compress the Disabled Period in Older Adults? J Am Geriatr Soc. 2016;64(10):1952-61.

6. Barnett K, Mercer SW, Norbury M, Watt G, Wyke S, Guthrie B. Epidemiology of multimorbidity and implications for health care, research, and medical education: a cross-sectional study. Lancet. 2012;380(9836):37-43.

7. Lafortune L, Martin S, Kelly S, Kuhn I, Remes O, Cowan A, et al. Behavioural Risk Factors in Mid-Life Associated with Successful Ageing, Disability, Dementia and Frailty in Later Life: A Rapid Systematic Review. PloS one. 2016;11(2):e0144405.

8. Koene RJ, Prizment AE, Blaes A, Konety SH. Shared Risk Factors in Cardiovascular Disease and Cancer. Circulation. 2016;133(11):1104-14.

9. Fried LP, Guralnik JM. Disability in older adults: evidence regarding significance, etiology, and risk. J Am Geriatr Soc. 1997;45(1):92-100.

10. Elwood P, Galante J, Pickering J, Palmer S, Bayer A, Ben-Shlomo Y, et al. Healthy lifestyles reduce the incidence of chronic diseases and dementia: evidence from the Caerphilly cohort study. PloS one. 2013;8(12):e81877.

11. Artaud F, Dugravot A, Sabia S, Singh-Manoux A, Tzourio C, Elbaz A. Unhealthy behaviours and disability in older adults: three-City Dijon cohort study. BMJ. 2013;347:f4240.

12. Newman AB, Arnold AM, Naydeck BL, Fried LP, Burke GL, Enright P, et al. "Successful aging": effect of subclinical cardiovascular disease. Arch Intern Med. 2003;163(19):2315-22.

13. Burke GL, Arnold AM, Bild DE, Cushman M, Fried LP, Newman A, et al. Factors associated with healthy aging: the cardiovascular health study. J Am Geriatr Soc. 2001;49(3):254-62.

14. Terry DF, Pencina MJ, Vasan RS, Murabito JM, Wolf PA, Hayes MK, et al. Cardiovascular risk factors predictive for survival and morbidity-free survival in the oldest-old Framingham Heart Study participants. J Am Geriatr Soc. 2005;53(11):1944-50.

15. Willcox BJ, He Q, Chen R, Yano K, Masaki KH, Grove JS, et al. Midlife risk factors and healthy survival in men. JAMA. 2006;296(19):2343-50.

16. Yates LB, Djousse L, Kurth T, Buring JE, Gaziano JM. Exceptional longevity in men: modifiable factors associated with survival and function to age 90 years. Arch Intern Med. 2008;168(3):284-90.

17. Franzon K, Byberg L, Sjogren P, Zethelius B, Cederholm T, Kilander L. Predictors of Independent Aging and Survival: A 16-Year Follow-Up Report in Octogenarian Men. J Am Geriatr Soc. 2017;65(9):1953-60.

18. Zhang S, Tomata Y, Discacciati A, Otsuka T, Sugawara Y, Tanji F, et al. Combined Healthy Lifestyle Behaviors and Disability-Free Survival: the Ohsaki Cohort 2006 Study. Journal of general internal medicine. 2019;34(9):1724-9.

19. Bosnes I, Nordahl HM, Stordal E, Bosnes O, Myklebust TA, Almkvist O. Lifestyle predictors of successful aging: A 20-year prospective HUNT study. PloS one. 2019;14(7):e0219200.

20. Swindell WR, Ensrud KE, Cawthon PM, Cauley JA, Cummings SR, Miller RA, et al. Indicators of "healthy aging" in older women (65-69 years of age). A data-mining approach based on prediction of long-term survival. BMC Geriatr. 2010;10:55.

21. McNeil JJ, Woods RL, Nelson MR, Reid CM, Kirpach B, Wolfe R, et al. Effect of Aspirin on Disability-free Survival in the Healthy Elderly. N Engl J Med. 2018;379(16):1499-508.

22. McNeil JJ, Wolfe R, Woods RL, Tonkin AM, Donnan GA, Nelson MR, et al. Effect of Aspirin on Cardiovascular Events and Bleeding in the Healthy Elderly. N Engl J Med. 2018;379(16):1509-18.

23. McNeil JJ, Nelson MR, Woods RL, Lockery JE, Wolfe R, Reid CM, et al. Effect of Aspirin on All-Cause Mortality in the Healthy Elderly. N Engl J Med. 2018;379(16):1519-28.

24. Group AI. Study design of ASPirin in Reducing Events in the Elderly (ASPREE): a randomized, controlled trial. Contemp Clin Trials. 2013;36(2):555-64.

25. Newman AB, Murabito JM. The epidemiology of longevity and exceptional survival. Epidemiol Rev. 2013;35:181-97.

26. Katz S. Assessing self-maintenance: activities of daily living, mobility, and instrumental activities of daily living. J Am Geriatr Soc. 1983;31(12):721-7.

27. Association AP. Diagnostic and statistical manual of mental disorders. Washington, DC: DSM IV; 1994.

28. van Buuren S, Groothuis-Oudshoorn K. mice: Multivariate Imputation by Chained Equations in R. Journal of Statistical Software. 2011;45(3):1-67.

29. S. VB. Flexible imputation of missing data. United Kingdom: Taylor & Francis Ltd; 2018.

30. White IR, Royston P. Imputing missing covariate values for the Cox model. Stat Med. 2009;28(15):1982-98.

31. White IR, Royston P, Wood AM. Multiple imputation using chained equations: Issues and guidance for practice. Stat Med. 2011;30(4):377-99.

32. Breheny P, Huang J. Group descent algorithms for nonconvex penalized linear and logistic regression models with grouped predictors. Stat Comput. 2015;25(2):173-87.

33. Friedman J, Hastie T, Tibshirani R. The elements of statistical learning. New York: Springer series in statistics; 2001.

34. Little RJA, Rubin DB. Statistical Analysis with Missing Data. Hoboken, NJ, USA: John Wiley & Sons, Inc.; 2002.

35. Thao LTP, Geskus R. A comparison of model selection methods for prediction in the presence of multiply imputed data. Biom J. 2019;61(2):343-56.

36. Blanche P, Dartigues JF, Jacqmin-Gadda H. Review and comparison of ROC curve estimators for a time-dependent outcome with marker-dependent censoring. Biometrical Journal. 2013;55(5):687–704.

37. Harrell FE. Regression modeling strategies: with applications to linear models, logistic and ordinal regression, and survival analysis. New York: Springer; 2015.

38. Musoro JZ, Zwinderman AH, Puhan MA, ter Riet G, Geskus RB. Validation of prediction models based on lasso regression with multiply imputed data. BMC Med Res Methodol. 2014;14:116.

39. Pahor M, Guralnik JM, Ambrosius WT, Blair S, Bonds DE, Church TS, et al. Effect of structured physical activity on prevention of major mobility disability in older adults: the LIFE study randomized clinical trial. JAMA. 2014;311(23):2387-96.

40. LaCroix AZ, LaMonte MJ, Applegate WB. The Long View of the LIFE Trial and a Life's Work. J Am Geriatr Soc. 2020;68(4):686-8.

41. Group SMIftSR, Williamson JD, Pajewski NM, Auchus AP, Bryan RN, Chelune G, et al. Effect of Intensive vs Standard Blood Pressure Control on Probable Dementia: A Randomized Clinical Trial. JAMA. 2019;321(6):553-61.

42. Guzman-Castillo M, Ahmadi-Abhari S, Bandosz P, Capewell S, Steptoe A, Singh-Manoux A, et al. Forecasted trends in disability and life expectancy in England and Wales up to 2025: a modelling study. Lancet Public Health. 2017;2(7):e307-e13.

**ASPREE INVESTIGATORS AND COMMITTEES**

**International Steering Committee**

John McNeil (Chair and Principal Investigator), Anne Murray (Co-Chair and Co-Principal Investigator), Lawrie Beilin, Andrew Chan, Jamehl Demons, Michael Ernst, Sara Espinoza, Matthew Goetz, Colin Johnston, Brenda Kirpach, Danny Liew, Karen Margolis, Frank Meyskens, Mark Nelson, Chris Reid, Raj Shah, Elsdon Storey, Andrew Tonkin, Rory Wolfe, Robyn Woods, John Zalcberg

**International End point Adjudication Committees**

Mark Nelson (Chair), Diane Ives (Co-Chair), Michael Berk, Wendy Bernstein, Donna Brauer, Christine Burns, Trevor Chong, Geoff Cloud, Jamehl Demons, Geoffrey Donnan, Charles Eaton, Paul Fitzgerald, Peter Gibbs, Andrew Haydon, Michael Jelinek, Finlay Macrae, Suzanne Mahady, Mobin Malik, Karen Margolis, Catriona McLean, Anne Murray, Anne Newman, Luz Rodriguez, Suzanne Satterfield (deceased), Raj Shah, Elsdon Storey, Jeanne Tie, Andrew Tonkin, Gijsberta van Londen, Stephanie Ward, Jeff Williamson, Erica Wood, John Zalcberg

**Data and Safety Monitoring Board**

Jay Mohr (Chair), Garnet Anderson, Stuart Connolly, Larry Friedman, JoAnn Manson, Mary Sano, Sean Morrison, Erik Magnus Ohman

**Australian Management Committee**

John McNeil (Chair), Robyn Woods (Deputy Chair), Walter Abhayaratna, Lawrie Beilin, Geoffrey Donnan, Peter Gibbs, Colin Johnston, Danny Liew, Trevor Lockett, Mark Nelson, Chris Reid, Nigel Stocks, Elsdon Storey, Andrew Tonkin, Rory Wolfe, John Zalcberg

**Publications, Presentations and Ancillary Studies Committee**

Anne Murray (Chair), Chris Reid (Co-Chair), Walter Abhayaratna, Michael Ernst, Colin Johnston, Beth Lewis, Danny Liew, Karen Margolis, John McNeil, Mark Nelson, Anne Newman, Thomas Obisesan, Raj Shah, Elsdon Storey, Robyn Woods

**International Data Management Committee**

Chris Reid (Chair), Jessica Lockery (Co-Chair), Michael Ernst, Dave Gilbertson, Brenda Kirpach, Raj Shah, Rory Wolfe, Robyn Woods

**ASPREE Data Management Center (Monash University) and Biostatistics**

Jessica Lockery (Data Manager), Taya Collyer, Jason Rigby; Programmers - Kunnapoj Pruksawongsin, Nino Hay; Biostatisticians – Rory Wolfe (Senior Biostatistician), Joanne Ryan, Kim Jachno, Catherine Smith; End point Processing – A.R.M.Saifuddin Ekram (Clinical Case Reviewer), Madeleine Gardam, Henry Luong, Tim Montgomery, Megan Plate, Laura Rojas, Anna Tominaga, Katrina Wadeson

**Australian Training, Recruitment, Retention and Operations Committee**

Suzanne Orchard (Chair), Sharyn Fitzgerald, Sarah Hopkins, Jessica Lockery, Trisha Nichols, Ruth Trevaks, Robyn Woods

**U.S. Operations/Recruitment and Retention Committee**

Brenda Kirpach (Chair), Ashley Johnson, Anne Murray, Molly Prozinski, Ramona Robinson-O’Brien, Nate Tessum

**SITE PRINCIPAL AND GP ASSOCIATE INVESTIGATORS**

**U.S. (2,411 participants):**

John Aloia, Steve Anton, Jeffery Burns, Gary Burton, Jamehl Demons, Charles Eaton*,* Michael Ernst, Sara Espinoza, Darron Ferris, Mahalakshmi Honasoge, Daniel Hsia, Steven Katzman, Anupama Kottam, Beth Lewis, Karen Margolis, Anne Murray, Shawna Nesbitt, Anne Newman, Thomas Obisesan, Augusto Ochoa, Pricilla Pemu, Kevin Peterson, James Powell, Gregg Pressman, William Robinson III, Susanne Satterfield (deceased), Raj Shah*,* Christine Thorburn*,* Elena Volpi, Jocelyn Wiggins, Jeff Williamson, Peter Wilson*,* Catherine Womack

**Australia (GP with at least one ASPREE participant; 16,703 participants):**

Abdullah M, Abdul-Ridha S, Aboud E, Abraham A, Abraham J, Abraham K, Abrahams M, Adad S, Adams C, Africa N, Afroze S, Agarwal D, Agbarakwe C, Ah Sang W, Ahern T, Ahmad Y, Ahmad Z, Ahmed L, Ajam A, Akhter R, Akram Z, Alagarswami K, Alam M, Alavi E, Aldridge L, Alethan A, Alexander K, Alexander L, Alexopoulos M, Ali B, Ali M, Allan J, Allen C, Allen G, Allen S, Allin P, Al-Musawy R, Alpren C, Al-Tawil I, Alwyn T, Amor P, Anam T, Anderson G, Anderson L, Anderson N, Anderson P, Anderson R, Anderson-Dalheim H, Andrada E, Andre S, Andrews L, Andric A, Andric M, Ang J, Ansari A, Arakji AM, Arambeploa Y, Ark R, Arnaudon FP, Arndt PM, Aroney T, Arthurson J, Arunachalam T, Asim N, Aslam I, Assad S, Astley N, Athari M, Atkins C, Atkins M, Aufgang M, Aung K, Aurora G, Auteri S, Avergun A, Awwad A, Azad C, Azra S, Babovic A, Baig M, Baker J, Baker S, Baker T, Bakhilova N, Baldam A, Baldassa A, Baldi C, Balkwill C, Balogun O, Ban A, Banerjee P, Banning M, Bansal S, Barkas R, Barker A, Barker D, Barnes A, Barnes N, Barnetson W, Barratt I, Barrett DA, Barrett Meagan, Barrett Michelle, Barrett P, Barrett T, Barson P, Barstad C, Barton W, Bartram M, Bartusek P, Basser S, Bassett S, Batchelor L, Batt D, Batty A, Baum S, Baxter M, Beaton G, Beaumont J, Beavis D, Beckett V, Beech M, Beilby J, Bekal S, Bell A, Bendtsen L, Benedict D, Benjamin T, Bennett P, Bennie G, Bennie S, Bennison S, Benson A, Benson R, Benson S, Bergin J, Bergin S, Berryman G, Berryman J, Bertram H, Bertuch G, Bettenay G, Bettiol L, Bills R, Birch J, Bird Rachel, Bird Robert, Birks R, Blake R, Blakney A, Blashki M, Bleach G, Bloch B, Bodenstein M, Boga V, Bollen C, Boltin P, Boon B, Booth G, Borg A, Bornstein D, Bottcher C, Bourke J, Bourke M, Boutcher S, Bowden J, Bowen J, Bowring B, Boyce C, Boyd J, Brack R, Bradshaw A, Brady P, Braithwaite J, Braude G, Brayshaw N, Breen M, Bresnahan R, Briddon P, Bridge A, Briggs SJ, Brimage RF, Britten-Jones W, Brkic M, Broadby M, Bromberger D, Brommeyer A, Broom I, Brophy T, Brough J, Brougham JP, Broun C, Brown ID, Brown J, Brown MB, Brown MP, Brown R, Brownbill C, Brownbill L, Browne M, Brownstein M, Bruce A, Brunacci F, Brunner C, Bruorton M, Buccheri V, Buchanan D, Buckley J, Bulle B, Bundy K, Burke M, Busch G, Bush CP, Butrev A, Bvirakare J, Bvumbura BF, Bye J, Byrne C, Byrne P, Cain M, Calcutt I, Calder K, Caldwell M, Callan C, Cameron A, Cameron David, Cameron Donald, Cameron T, Campbell David, Campbell Donald, Campbell Geoffrey, Campbell Guy, Campbell PH, Campbell R, Carroll N, Carroll V, Carson J, Carson R, Carter L, Carter P, Carter R, Carter S, Cartwright P, Cassidy P, Catchpole M, Cato G, Celada R, Chai F, Chalabi A, Chalissery P, Chalmers ML, Chamberlain H, Chamoun R, Chan B, Chan C, Chan CK, Chan FW, Chan K, Chandran S, Chandrananth M, Chandrananth S, Chang C, Chang V, Chang W, Changakoti A, Chantler R, Chao D, Chao S, Charlton P, Chattersee A, Chau G, Chaung Y, Chawtur V, Cheah H-H, Cheah S, Cheasley A, Chee H, Chen D, Cheng W, Chesney D, Chew D, Chhabra P, Chia I, Chia P, Chiang A, Chiang S, Chiew I, Chiew L, Chikarsal A, Chin J, Chin M, Chipman JS, Chipperfield C, Chisholm H, Chisholm L, Chiu A, Chiu C, Chiu D, Chiu T, Chizik L, Choksey H, Choo E, Chow Amy, Chow Andrew, Choy C, Chu S, Chua A, Chuah T, Chung J, Cimpoescu T, Clapton J, Clark Benedict, Clark Benjamin, Clark M, Clark R, Clarke A, Clarke D, Clarke S, Cleary G, Clerigo L, Clohesy S, Close S, Cochrane F, Cohen IS, Cohen J, Colahan R, Collins J, Colman W, Colvin R, Conde S, Connell P, Connellan M, Connor W, Connors G, Conos M, Conron D, Conroy J, Conway C, Cooper M, Cooper S, Cope A, Corrigan Simon, Corrigan Sue, Coughlan P, Coulter E, Counsel L, Court D, Courtis G, Cousens A, Craig L, Crameri M, Cranswick M, Crawford J, Crawford M, Crawford P, Crawford R, Crick S, Crimmins B, Cristofaro R, Croatto J, Crompton A, Cronin E, Crookes J, Cross B, Cross D, Cross M, Crow P, Crowe JE, Crowe P, Crowley H, Cruickshank J, Cummins R, Cunneen A, Cunningham A, Cunningham N, Cunningham P, Curnow D, Curran J, Curran M, Currie A, Curtis R, Cusack J, Dabash K, Dabestani V, Dadabhay Z, Daglas D, Dagley P, Danesh S, Dang D, Daniels R, Darby JP, Darko N, Darling J, Darlington B, Das J, Das P, Date M, Datta C, Datta S, Davenport C, Davey G, Davey M, Davey P, Davidson CL, Davidson D, Davies M, Davies-Hakeem A, Davis G, Davis K, Davis Paul, Davis Peter, Davis S, Dawe N, Dawes R, Dawkins P, Dawson G, Dawson P, Dawson R, Day P, Daya M, Dayasagar D, D’Costa L, De Clifford M, De Gleria S, De Poi C, De Silva M, De Silva P, De Steiger R, De Villiers D, De Wit E, Debnath R, Deery R, DeL anerolle D, Del Rio F, Delaney S, Delitzsch SS, Demaio F, Demian M, Demirtzoglou J, Denton T, Derrick L, Deshmukh K, Dessauer J, Devavittiya C, Devereux D, Dewan D, Dewhurst H, Dhar A, Dhillon D, Di Carlo M, Di Dio A, Di Marco A, Dickman J, Dillon L, Dinh Q-T, Dissanayake D, Dissanayake M, Dissanayake T, Divakaran K, Dixit U, Dixon H, Dixon N, Djakic E, Dobson C, Dodd L, Dodds P, Dodic A, Dodic M, Doley A, Dolguina S, Dolling C, Donaghy F, Donald H, Donelan E, Donohue M, Dooland J, Dooley H, Doslo S, Douglas A, Dover P, Downe G, Drake P, Dry D, Duane P, Dubash A, Dubetz D, Duff P, Duke R, Dumitrescu C, Dunbar A, Dunbar S, Dunn S, Duong NH, Dutta N, Dutton M, Duval A, Dyson-Berry J, Eade P, Eaton D, Ebert K, Edib K, Edillo E, Edmonds J, Edwards F, Edwards PA, Edwards S, Eftekharuddin M, Egan A, Egan P, Ehrenreich S, Ehsan E, Elberg L, Elisha B, Elisha R, Elkhoury H, Ellerton K, Elliot-Smith A, Elmore R, Elshenawy I, Elsherif S, Elsouki M, Elton P, Emmerson M, Emmett SI, English J, Enten P, Entwistle J, Epa W, Erhardt A, Etta J, Evans M, Everitt T, Ewing J, Fahkok B, Faigen M, Fair A, Fairbrother C, Fanning J, Fantasia M, Farag E, Fardell K, Farrant J, Farrell P, Farrow J, Fassett M, Faull PA, Ferguson P, Fernando Sujeewa, Fernando Sumudu, Ferruccio A, Fidge JH, Field P, Figurireo L, Fisher H, Fisher J, Fitzgerald E, Fitzgerald M, Fitzgerald R, Fitzpatrick H, Fitzpatrick J, Fitzpatrick P, Fitzpatrick T, Flaherty P, Flanagan D, Flanagan T, Flew S, Fonseka PP, Foo J, Foo S, Foo Y, Foong E, Ford D, Foster D, Fourlanos V, Fowler I, Fox D, Fox F, Fox M, Fox P, Fox-Smith D, Francis J, Francis R, Frank O, Franks A, Fredericks A, Freeman E, French L, Frew B, Friebel D, Friebel T, Frost S, Fryer D, Fuller J, Fung W, Fung WP, Furphy S, Gabutina C, Gaggin S, Galbraith S, Gale M, Gall J, Gallichio V, Gangell AW, Garde MA, Gardner SS, Gardner T, Garland J, Garra G, Garrow S, Garvey J, Gauden M, Gault A, Gaur D, Gavralas A, George N, George S, Georgy M, Gerendasi R, Geschke H, Giannakakis J, Gidley G, Gilani M, Giles P, Gill K, Gill P, Gill R, Gillis C, Gilmore A, Gilovitz M, Gingold R, Glaspole D, Glowinski L, Glue AL, Godakumbura P, Godavarthy R, Goel A, Goeltom C, Goldberg E, Goldberg J, Golets M, Gong V, Goode J, Goodman C, Goodwin RJ, Gopathy S, Gordon M, Gough S, Govender M, Gow K, Gowrie B, Goy P, Grabowski C, Graddon J, Granek A, Gray JM, Gray M, Gray T, Grbac E, Greacen J, Greculescu E, Green J, Greenwood E, Griffin E, Griffith V, Griffiths A, Griffiths G, Griffiths J, Griffiths K, Grigorian AR, Grinzi P, Grogan H, Grokop G, Grossman L, Grove A, Gruzauskas A, Gu M, Guest S, Guindi N, Guo H, Gurney R, Guy J, Guymer J, Gwynn R, Gyorki J, Habibi S, Hachem C, Hackett A, Hackett J, Haddad J, Haddad M, Hadley E, Hagger R, Haider Z, Hain R, Hajicosta T, Hales P, Hall J, Hall P, Hall Robert, Hall Roslyn, Hall S, Halliburton K, Halliday A, Halliday B, Halliday J, Hamblen K, Hamel J, Hamer I, Hamilton J, Hamilton RF, Hammond T, Hanbury R, Hancock A, Hand R, Hanna A, Hanna M, Hanna S, Hanson G, Hanson PD, Haque E, Haran C, Haran T, Hare WJ, Harewood A, Haripersad S, Harman A, Harmer D, Harms P, Harnden C, Harrington M, Harris A, Harris M, Harrison M, Harrison S, Hart E, Hartley D, Hartley P, Hartnett M, Harvey C, Haslam K, Hassani I, Hassett RB, Hastings W, Hattingh A, Hawke I, Hawkins C, Hayes V, Heale J, Healy G, Hebblewhite A, Hechtman A, Hedgland A, Heffernan C, Heikkinen MN, Heinrich C, Henderson J, Henry F, Herath S, Herbert A, Herbst D, Hermiz S, Herrman J, Hesse M, Hetherington J, Hetzel R, Hewett R, Hides R, Higgins CD, Hildred S, Hill A, Hilton C, Hince R, Hines C, Hinton C, Hipolito A, Ho CK, Ho L, Hoar J, Hocking L, Hodge A, Hodgkins A, Hodgson J, Hogbin J, Hok S, Holder B, Holland D, Holland M, Hollins B, Homewood M, Hong Zhou A, Honig J, Honigman S, Hookham D, Hooper W, Hope L, Horman J, Horng T, Hornstein I, Horriat M, Horvat J, Hossain M, Hough P, Howe J, Howson W, Hubczenko I, Hubel M, Hughes J, Hughes P, Hunter D, Huq S, Hussain A, Hutchins I, Hutchinson A, Hyam P, Hyare K, Iakovidis B, Ibragimov M, Idris M, Ierace C, Ikladios A, Imgraben P, Ingham C, Ip A, Ip Y, Iqbal A, Iqbal M, Irvine G, Irwin V, Iser D, Islam N, Islam S, Isles JK, Ismail A, Ivanoff G, Iwe N, Jackett RB, Jackson M, Jackson N, Jackson P, Jackson T, Jacoup M, Jaensch E, Jain P, Jain S, Jaiswal N, Jaksic A, Jakubowicz I, Jamel B, James J, Jameson D, Jansz C, Jarman E, Jassi I, Jayasinghe S, Jayatilake J, Jayaweera V, Jeanes R, Jeanneret CI, Jedynak S, Jeffries L, Jegadeesh K, Jenkins P, Jennings C, Jenny C, Jiang YY, Jigau C, Jinadasa C, Joel S, John R, Johns P, Johnson C, Johnson J, Johnson M, Johnson N, Johnson W, Johnston B, Johnston K, Johnston M, Johnston R, Johnston T, Jones G, Jones I, Jones L, Jones M, Jones S, Jones Tania, Jones Tudor, Joshi M, Joshi Naveen, Joshi Nirupama, Joske F, Joubert C, Jovanovic B, Joyce R, Judd AM, Judd J, Kaaden JP, Kabat L, Kabourakis F, Kaippilly A, Kajani H, Kamale A, Kaminsky L, Kanapathipillai U, Kanashuk L, Kao R, Kapadia P, Kapadia V, Karmouche R, Kaur KJ, Kavanagh T, Kay A, Kay B, Kaye S, Keane K, Keating B, Keecha E, Keecha J, Keenan P, Keillar P, Kemp G, Kemp P, Kennedy M, Kennedy U, Kennett S, Kesarapu S, Khan F, Khan I, Khan M, Khong CK, Khoo F, Khoo J, Khoo S, Khoshghalb A, Kiefer J, Kiley M, Kilov G, Kimpton N, King SC, Kingston R, Kinsella P, Kipouridis A, Kirwan A, Kisselev S, Kitchen J, Kloot S, Knaggs J, Knight E, Knobel J, Knowles D, Knowles P, Kogosowski S, Kok Jereth, Kok Joyce, Kollios D, Konopnicki H, Koravos A, Korol P, Kosky AR, Kote Somashekarappa M, Kottegoda-Vithana E, Kotur S, Kozminsky M, Kraner G, Kraus DH, Krell I, Kruytbosch C, Kuay V, Kucminska A, Kulatunga P, Kulinski M, Kumar J, Kumar R, Kumar S, Kumarage D, Kumaraswamy S, Kunze M, Kurien S, Kuruvilla P, Kwong R, Kyaw Z, Kyriacopoulos J, Lackner PJ, Lahanis C, Lajoie D, Lajoie K, Lakshmanan A, Lal A, Lalor E, Lam D, Lambooij C, Lancaster M, Landa L, Landers J, Lane R, Langston K, Lapin S, Lath P, Lau-Gooey T, Lawlor-Smith L, Le Couteur S, Le P, Le Riche M, Le V, Le W, Leber D, Ledner A, Lee B, Lee C, Lee D, Lee FB, Lee Jade, Lee James, Lee Jessicasu-Yin, Lee John, Lees K, Lees R, Lees W, Leffler P, Lenton J, Leong R, Leow L, Leow P, Leow Y, Leslie N, Lester SE, Lewi L, Lewis P, Lewis R, Li A, Li J, Liang J, Liang Xs, Libhaber H, Lichtblau B, Lickiss T, Liedvogel M, Liew K, Light L, Lightfoot W, Lim C, Lim D, Lim H, Lim HS, Lim J, Lim SG, Limaye S, Limbu Y, Lindenmayer J, Lindstedt P, Lines A, Ling J, Ling R, Linton J, Linton S, Linton T, Liow C, Liow YC, Lip L, Lipson D, Liu S, Liu Y, Liubinas R, Liveriadis T, Lizner S, Lloyd M, Lo B, Lo C, Lock P, Lockhart M, Logan M, Loke KP, Long Matthew, Long Michael, Longworth W, Loo KH, Lopez-Hernandez S, Lord RJ, Louw J, Louw TT, Low B, Low F, Lowe M, Lowther D, Loxley P, Lu P, Lu S, Lucarelli A, Lui G, Lui K, Lui R, Luke C, Lukic N, Lupton J, Luscombe T, Luttrell CL, Lyall A, Lynch J, Lynn K, Lyon D, Lyon E, Lyons S, Macaulay G, Macaulay K, MacIndoe A, MacIsaac P, Maciver R, Mackay B, Mackay J, Mackinnon D, Mackle R, Macphail J, Madawala N, Madden J, Madeley C, Madhanpall N, Magarey J, Magill M, Mah S, Mahadeva SP, Mahendran S, Maher J, Maher M, Mahmood Aamir, Mahmood Abbas, Maier K, Majchrzak W, Majeed J, Makar A, Makohon R, Malcher P, Malcolm HE, Malcolm M, Mallett S, Mallik A, Manderson J, Mane S, Mangan G, Manifold M, Manoliadis M, Manovel B, Mansour A, Manton D, Marano F, Marchant D, Mariajoseph G, Marinos A, Marinucci D, Marrows M, Marsh D, Martin C, Martin G, Martin R, Marton F, Martynova L, Mason N, Masood U, Massaud M, Massy- Westropp P, Masters B, Mather J, Mathews RA, Mathieson G, Mauro M, Mauviel PA, Maxfield N, Mayhead C, Mazengiya S, Mazhar A, Mbachilin G, McAllan A, McCallum H, McCann N, McCarthy A, McCleary A, McClelland R, McConville DS, McCorkell J, McCormack G, McCormick H, McCowan M, McCutcheon J, McDonald AG, McDonald AS, McDonald IR, McDonald J, McDonald N, McDonald S, McEniery A, McEntee K, McGee R, McGinity P, McGowan N, McGowan R, McGrath L, McGuire Paul, McGuire Precious, McHardy C, McHenry K, McIllree R, McKay M, McKellar C, McKelvie M, McKenzie SI, McKeown J, McKeown M, McKernan S, McKinnon A, McLaren G, McLeod I, McMahon A, McMaster I, McNab NR, McNaughton EL, McNiff M, McPherson C, Meaney J, Medlicott M, Medres R, Megally R, Mehta K, Mellios O, Melvani R, Mencel J, Mendick S, Mendis L, Menzies J, Mercado M, Mesiha S, Meyer PL, Meyer R, Miceli A, Michaelson T, Michail A, Michelmore K, Miezis V, Milan S, Milky S, Miller K, Milner J, Milone R, Milton C, Milward N, Mirhom R, Mirranay S, Mishricky H, Misso R, Mitchell A, Mitchell D, Mitchell L, Mobilia G, Moffitt M, Mohr V, Moller Gary, Moller Graeme, Molloy P, Molloy T, Molyneux P, Monaghan C, Monash D, Moncrieff S, Monzon M, Mooney T, Moore E, Moran J, Morgan G, Morgan M, Morgan N, Morris N, Morris S, Morrison H, Morrow S, Morton R, Moschou C, Moulding S, Moule V, Mouzakis V, Mudunna D, Mudzi S, Mulkearns P, Mullen D, Mulvey G, Mungi D, Munro L, Muraledaran S, Murphy B, Murphy G, Murray A, Murray B, Murray E, Murray H, Murray S, Murtagh C, Nadarajah M, Naiker S, Naing W, Nandha R, Nankervis J, Naoum A, Nash C, Nashed M, Nasreen N, Nath-Chand U, Neagle M, Nelson C, Nelson MR, Nesbitt P, Neuberger M, Newman S, Newton S, Ng D, Ng H, Ng S, Nguyen D, Nguyen HQ, Nguyen HT, Nguyen T, Nguyen-Ngoc M, Nice P, Nicholls P, Nicholson D, Nicola N, Nicolettou N, Nicolson I, Nield S, Nikolic V, Nikolovska-Buzevski N, Nilsson A, Nimmo A, Nisselle P, Nitchingham S, Niven A, Nnopu E, Noonan L, Norton C, Norton G, Notini G, Nwaegerue ED, Nylander P, O’Brien C, O’Connor A, O’Connor DA, O’Donovan B, O’Driscoll E, Oechsle G, Offor J, Ogilvie B, O’Halloran J, O’Hanlon P, Okolie K, Olaniyi I, O’Leary B, O’Leary K, Olesen J, Oliver P, Olomola O, Olszewski C, Olukolu G, Omarjee A, Omidiora AA, Omifolaji S, O’Neill A, O’Neill CO, Ong BP, Ong M, Ooruthiran M, Oppermann BL, Orbach E, Orgonas R, Orsillo M, Ostberg M, O’Sullivan C, O’Sullivan J, O’Sullivan PJ, O’Toole C, O’Toole M, Otuonye D, Owen T, Padilla C, Page A, Pahuja P, Palmer A, Pan J, Panozzo D, Pantillano E, Papagelis A, Papas E, Pape A, Paransothy P, Parghi N, Parker A, Parker J, Parker S, Parkes H, Parletta E, Parry B, Pasha M, Patel G, Patel M, Pathirana A, Patterson R, Pattichis I, Pattison J, Pava C, Peachey D, Pearce E, Pearce R, Pearse B, Pearson R, Pech M, Peduru-Arachchige A, Pellegrini P, Pellizzari G, Pereira V, Perera B, Perera L, Perlesz A, Perraton R, Perry H, Perry S, Perry W, Pervaiz Z, Peters L, Pham H, Phan C, Phan T, Phare A, Philip J, Philips J, Phillips A, Philpot J, Phiri R, Pickavance M, Piekarski D, Pienkos J, Piez W, Pilgrim C, Pillai BK, Pinder R, Pinkstone J, Pinson J, Pither A, Plenderleith J, Pliatsios B, Plunkett M, Pokharel C, Poland D, Polgar V, Polmear D, Poologanathan G, Pope I, Popp L, Portelli A, Potter T, Powell Kendra, Powell Kristine, Powell V, Power R, Powles A, Poynton N, Pranavan S, Prasad R, Praszkier S, Preiss J, Pretorius P, Price C, Price I, Price K, Price M, Priest C, Pring M, Profitt C, Protassow A, Psaradellis IJA, Psycharis J, Pucilowski D, Pun K, Qamar F, Quach S, Radcliff E, Radcliffe B, Radcliffe J, Radford J, Ragg P, Rahel E, Rahim T, Rahman F, Rahmanamlashi N, Rajasooriar S, Rajendra I, Rajini E, Raman A, Ramsay A, Ramsey J, Rana U, Rankin M, Rao UV, Rapley M, Rasaratnam S, Rashid A, Ratnaike L, Rattan J, Ratten K, Rattraywood C, Rayner E, Rea J, Rea PC, Reddy Sanganakal, Reddy Shradhanand, Reed R, Reeves C, Reichl T, Reid J, Reid K, Remyn P, Renfrey S, Renouf E, Renshaw P, Retchford A, Reynolds F, Reza R, Rezk L, Rhee J, Rhodes F, Rice A, Richards J, Richards R, Richardson A, Richardson GT, Richardson R, Richardson T, Ridgers D, Ridgers MJ, Rieger W, Rienits H, Rigoni M, Riley J, Rillstone D, Rimmer DE, Ringelblum D, Riseley J, Roberts A, Roberts I, Roberts J, Roberts M, Roberts S, Robinson J, Robinson R, Robson A, Roche V, Rodda C, Rodway P, Roebuck R, Rogers D, Rogers S, Roman F, Romas D, Ronan C, Rope S, Rose A, Rose DF, Rose G, Rose K, Rosen N, Rosenblatt J, Ross K, Ross Mary, Ross T, Roth J, Rothfield J, Roubos N, Roufael AD, Rounsevell J, Rouse W, Roushdy B, Rowe R, Rowland G, Roy A, Royston A, Rubin J, Russell G, Ryan F, Ryan N, Ryan S, Sabet A, Sabetypeyman F, Sachdev A, Saddik A, Sadhai R, Saeed S, Sahhar C, Saka M, Salauddin M, Salter E, Salter M, Samaddar A, Samarakkody A, Samararatna M, Samarsekera C, Samuel-John D, Sandars M, Sanders J, Sanderson L, Sandhu N, Sandrasegaram S, Sangsari A, Saprid J, Sarkis K, Sasse C, Satter F, Satyadharma K, Saul J, Scaife R, Schaap M, Scheelings FT, Schinckel H, Schlesinger P, Schlicht S, Schmidt M, Schneeweiss A, Schroeder E, Scully S, Searle R, Sebastian T, Seeto R, Segal G, Segal L, Seidel B, Selga A, Senanayake I, Seneviratne M, Seneviratne T, Senini D, Senior J, Seow L, Sepetavc D, Serafim A, Serban R, Sexton P, Shahat M, Shamoun Y, Shanmugarajah K, Shannon G, Sharif A, Shariff A, Sharma A, Sharma D, Sharma M, Sharma P, Sharma R, Sharma S, Sharma U, Sharp V, Sheen-Apostol J, Sheikh Mohamed M, Sher J, Sherley M, Shi B, Shimmin MB, Shing D, Shires SE, Shmerling A, Shortis P, Shroot AD, Shute J, Sia M, Siapantas S, Sidhwarni R, Siemienowicz J, Siew HC, Sigalov E, Silver D, Simes L, Simonson F, Simpson R, Simpson T, Simpson W, Singh B, Singh D, Singh H, Singh M, Singh R, Siow CL, Sitlington R, Sivapalan C, Skeat J, Skehan M, Skeklios L, Skinner T, Sklovsky CJ, Slabbert J, Slaney GM, Slattery C, Sleaby E, Sleiman C, Slesenger J, Slimming T, Sloan C, Sloane R, Slonim D, Slot P, Smagas T, Smart M, Smibert L, Smiley J, Smith D, Smith G, Smith J, Smith P, Smith R, Smith Stephen, Smith Stuart, Smith V, Smylie D, Sneyd S, Snow S, Sobol G, Soccio M, Solanki V, Soloczynskiyj A, Solomon D, Somerville M, Song J, Soo D, Soo L, Soo T, Soo TM, Sood R, Sooknandan S, Soon M, Sosnin M, Spanos N, Spargo JS, Speirs B, Spencer H, Spencer J, Spottiswood M, Spring M, Squires L, Stabelos G, Stagg M, Stanley L, Stark A, Steel A, Steer N, Steiner H, Stephanson A, Stephens G, Stephenson A, Sterling BR, Stevens B, Stevens P, Stevenson J, Stewart C, Stewart R, Sticklen E, Stiebel P, Stillger JM, Stinerman I, Stobie M, Stobie T, Stojkovski S, Stone A, Stowe S, Stoyanova V, Strasser K, Strong J, Struk H, Stuart A, Su J, Sujecki M, Suka R, Sullivan T, Sululola A, Sumathipala A, Suntesic L, Sutherland D, Sutherland I, Sutherland R, Sutton J, Swart R, Sweet M, Sweet R, Syed Z, Sykes J, Sylivris A, Symon B, Szabo R, Sze J, Szenczy C, Sze-Tho R, Szymanski I, Szymanski R, Tadrous M, Taft D, Taine M, Talic D, Tan Elaine, Tan Eng, Tan G, Tan HM, Tanovic A, Tasiopoulos A, Tate K, Tattersall I, Taverna C, Taylor J, Taylor R, Taylor S, Teo K, Teoh C, Teperman B, Tereszkiewicz W, Thanenthiran R, Thangarajah C, Thangavel B, Thann Z, The S, Theophilos M, Theris N, Thiru K, Thiru M, Thomas G, Thomas P, Thompson D, Thompson L, Thompson W, Thomson B, Thorne A, Thornley J, Thorpe V, Thottakurichi R, Thurairajah A, Thurairajah S, Thyagarajan T, Tiet Q, Tillekeratne K, Tine S, Tinning R, Tinston C, To E, Tolentino C, Tom H, Tomar D, Tomic M, Tomyn L, Toohill G, Tooth M, Tormey S, Toua P, Trainor S, Tran C, Tran E, Tran LD, Tran TQ, Trethowan K, Trevena R, Trigg P, Trivett B, Try R, Tsigopoulos A, Tucker D, Tunaley S, Turnbull H, Turnbull S, Turner J, Twycross W, Tynan D, Tyndall P, Tyshing W, Uchendu F, Uhlenbruch B, Uluca U, Unkenstein D, Urie JP, Vaiopoulos T, Van Ammers E, Van Der Merwe D, Van Der Spek A, Van Der Vlist R, Van Opstal E, Vanderzeil G, Vanderzeil T, Vanker L, Vanmali H, Varghese A, Varney W, Vasquez I, Vasudevan S, Veal M, Venables S, Venkatram G, Verghese P, Verma H, Verma R, Verso M, Victor A, Vijayakumar V, Vijayanand P, Viljoen E, Vincent F, Vinci A, Vinci G, Viney P, Visvalingam C, Von Caemmerer A, Vonschmidt JK, Vorich R, Vrij R, Vyas S, Wai T, Waid S, Wakefield B, Walder D, Waldron CM, Waldron M, Wales S, Walker B, Walker G, Walker R, Walker W, Wall R, Wallace J, Wallace K, Wallis I, Wang S, Wang X, Wang Z, Ward C, Ward R, Ward S, Wardlaw P, Wark A, Warr A, Warren M, Waters L, Watson A, Watson S, Watt G, Watt J, Watterson J, Waugh R, Wazid M, Wearne E, Webb I, Webber C, Webber E, Webber S, Webster DL, Webster J, Webster Peter, Webster Philip, Weerasinghe S, Weerasoorya M, Weinrich J, Welberry L, Weller A, Wells S, Welsh D, Weng M, Wenig M, Wettesinghe I, Wexler P, White A, White G, White Roxana, Whitehouse J, Whitehouse L, Whitehouse R, Whitfield K, Whitfield S, Whitney W, Wiehle G, Wight R, Wild I, Wilding S, Wildman G, Williams A, Williams G, Williams J, Williams M, Williams PD, Williams S, Williams W, Willis M, Wilson A, Win N, Wiseman J, Wishart W, Wivell F, Wong C, Wong CS, Wong D, Wong John K, Wong Johnny, Wong Ju-Min, Wong P, Wong PT, Wong Y, Wood P, Woods R, Woodward P, Wooff D, Woolf S, Worboys P, Worboys PC, Wrennall R, Wright Adrian, Wright Antony, Wright L, Wright Richard, Wright Robert, Wrobel K, Wu D, Wu E, Wu L, Xiao M, Yacoub M, Yang A, Yang J, Yang R, Yates D, Yazbek P, Yeaman C, Yeo M, Yeung Shi Chung D, Yiap D, Yilmaz S, Yogaranandan D, Young D, Young R, Young S, Yousef M, Yousif K, Youssef D, Yu Z, Yuille R, Zagorksi M, Zail S, Zain M, Zallmann A, Zeng L, Zhao S, Zhao W, Zheng M, Zhou D, Ziccone M, Zimmerman J, Zwijnenburg A.

**Tables and Figures**

Table 1: Baseline characteristics of the trial population

|  | **All participants** | | **Male** | | **Female** | |
| --- | --- | --- | --- | --- | --- | --- |
| **Characteristic** | **N** | **N = 19,114** | **N** | **N = 8,332** | **N** | **N = 10,782** |
| Age (years) | 19,114 | 74.0 (71.6, 77.7) | 8,332 | 73.8 (71.6, 77.3) | 10,782 | 74.1 (71.7, 77.9) |
| Ethnicity/Race | 19,114 |  | 8,332 |  | 10,782 |  |
| White |  | 17,450 (91.3%) |  | 7,681 (92.2%) |  | 9,769 (90.6%) |
| Black |  | 901 (4.7%) |  | 307 (3.7%) |  | 594 (5.5%) |
| Hispanic |  | 488 (2.6%) |  | 202 (2.4%) |  | 286 (2.7%) |
| Others |  | 275 (1.4%) |  | 142 (1.7%) |  | 133 (1.2%) |
| Country | 19,114 |  | 8,332 |  | 10,782 |  |
| Australia |  | 16,703 (87.4%) |  | 7,524 (90.3%) |  | 9,179 (85.1%) |
| US |  | 2,411 (12.6%) |  | 808 (9.7%) |  | 1,603 (14.9%) |
| Living status | 19,114 |  | 8,332 |  | 10,782 |  |
| At home alone |  | 6,251 (32.7%) |  | 1,729 (20.8%) |  | 4,522 (41.9%) |
| At home with family, friends, or spouse |  | 12,863 (67.3%) |  | 6,603 (79.2%) |  | 6,260 (58.1%) |
| Years of education | 19,113 |  | 8,332 |  | 10,781 |  |
| < 9 |  | 3,002 (15.7%) |  | 1,347 (16.2%) |  | 1,655 (15.4%) |
| 9-11 |  | 5,634 (29.5%) |  | 2,306 (27.7%) |  | 3,328 (30.9%) |
| 12 |  | 2,319 (12.1%) |  | 947 (11.4%) |  | 1,372 (12.7%) |
| 13-15 |  | 3,255 (17.0%) |  | 1,344 (16.1%) |  | 1,911 (17.7%) |
| 16 |  | 1,766 (9.2%) |  | 734 (8.8%) |  | 1,032 (9.6%) |
| 17-21 |  | 3,137 (16.4%) |  | 1,654 (19.9%) |  | 1,483 (13.8%) |
| Type 2 Diabetes | 19,114 | 2,045 (10.7%) | 8,332 | 1,046 (12.6%) | 10,782 | 999 (9.3%) |
| Smoking history | 19,114 |  | 8,332 |  | 10,782 |  |
| Current |  | 735 (3.8%) |  | 383 (4.6%) |  | 352 (3.3%) |
| Former/Never |  | 18,379 (96.2%) |  | 7,949 (95.4%) |  | 10,430 (96.7%) |
| Alcohol consumption | 19,114 |  | 8,332 |  | 10,782 |  |
| Current |  | 14,642 (76.6%) |  | 6,932 (83.2%) |  | 7,710 (71.5%) |
| Former/Never |  | 4,472 (23.4%) |  | 1,400 (16.8%) |  | 3,072 (28.5%) |
| Family history of myocardial infarction | 17,605 | 497 (2.8%) | 7,674 | 180 (2.3%) | 9,931 | 317 (3.2%) |
| History of cancer | 19,035 | 3,660 (19.2%) | 8,294 | 1,790 (21.6%) | 10,741 | 1,870 (17.4%) |
| Non-HDL-c (mmol/L) | 18,666 | 3.60 (3.00, 4.30) | 8,125 | 3.60 (3.00, 4.20) | 10,541 | 3.60 (3.00, 4.30) |
| HDL-c (mmol/L) | 18,668 | 1.50 (1.30, 1.80) | 8,126 | 1.30 (1.10, 1.60) | 10,542 | 1.70 (1.40, 2.00) |
| eGFR (mL/min per 1.73 m^2^) | 18,650 | 74 (63, 84) | 8,114 | 74 (64, 84) | 10,536 | 74 (63, 85) |
| Haemoglobin (g/dL) | 19,112 | 14.10 (13.30, 15.00) | 8,330 | 14.90 (14.10, 15.60) | 10,782 | 13.60 (13.00, 14.30) |
| Systolic blood pressure (mmHg) | 19,114 | 139 (127, 151) | 8,332 | 141 (130, 152) | 10,782 | 137 (125, 149) |
| Diastolic blood pressure (mmHg) | 19,114 | 77 (70, 84) | 8,332 | 78 (72, 85) | 10,782 | 76 (69, 84) |
| Body-mass-index (kg/m^2^) | 19,025 | 27.5 (24.9, 30.7) | 8,300 | 27.6 (25.3, 30.1) | 10,725 | 27.5 (24.5, 31.2) |
| Waist circumference (cm) | 18,905 | 97 (89, 105) | 8,269 | 101 (95, 108) | 10,636 | 93 (84, 102) |
| Grip strength (kg) | 18,828 | 25 (20, 34) | 8,231 | 35 (30, 40) | 10,597 | 20.7 (17.0, 24.3) |
| Gait speed (m/s) | 19,018 | 1.01 (0.87, 1.15) | 8,295 | 1.04 (0.91, 1.19) | 10,723 | 0.99 (0.84, 1.13) |
| Lipid-lowering agents | 19,114 | 5,987 (31.3%) | 8,332 | 2,337 (28.0%) | 10,782 | 3,650 (33.9%) |
| Antihypertensive agents | 19,114 | 10,062 (52.6%) | 8,332 | 4,089 (49.1%) | 10,782 | 5,973 (55.4%) |
| Treatment randomization | 19,114 |  | 8,332 |  | 10,782 |  |
| Placebo |  | 9,589 (50.2%) |  | 4,180 (50.2%) |  | 5,409 (50.2%) |
| Aspirin |  | 9,525 (49.8%) |  | 4,152 (49.8%) |  | 5,373 (49.8%) |
| 3MS | 19,114 | 94 (91, 97) | 8,332 | 94 (90, 96) | 10,782 | 95 (92, 97) |
| CES-D (≥8) | 19,110 | 1,879 (9.8%) | 8,331 | 631 (7.6%) | 10,779 | 1,248 (11.6%) |

The numbers are presented as median with inter-quartile-range or as absolute and relative numbers. Abbreviations: N = Number of participants with available data, HDL-c = high-density-lipoprotein-cholesterol, 3MS = Modified Mini-Mental State, CES-D = Centre for Epidemiologic Studies-Depression 10 question assessment.

Table 2: Multivariable Cox regression analyses based on the selected predictors of disability-free survival. Estimates were pooled across multiple imputed data sets using Rubin’s rule.

|  | **Male** | | | **Female** | | |
| --- | --- | --- | --- | --- | --- | --- |
| **Characteristic** | **HR** | **95% CI** | **p-value** | **HR** | **95% CI** | **p-value** |
| Age (+5 years) | 1.49 | 1.38, 1.60 | <0.001 | 1.50 | 1.40, 1.61 | <0.001 |
| 3MS (+5 units) | 0.74 | 0.69, 0.79 | <0.001 | 0.68 | 0.63, 0.72 | <0.001 |
| Gait speed (+0.2 m/s)* | 0.82 | 0.77, 0.88 | <0.001 |  |  | <0.001 |
| 0.5 vs. 1.0 m/s | 1.64 | 1.38, 1.95 |  | 2.71 | 2.20, 3.33 |  |
| 1.5 vs. 1.0 m/s | 0.61 | 0.51, 0.73 |  | 0.80 | 0.56, 1.15 |  |
| Grip strength | 0.92 | 0.88, 0.96 | <0.001 | 0.91 | 0.85, 0.97 | 0.003 |
| Current smoking | 1.95 | 1.51, 2.52 | <0.001 | … | … | … |
| BMI (kg/m^2^)* |  |  | <0.001 |  |  | <0.001 |
| 25 vs. 30 kg/m^2^ | 1.15 | 1.05, 1.26 |  | 1.11 | 1.03, 1.19 |  |
| 35 vs. 30 kg/m^2^ | 1.17 | 1.02, 1.35 |  | 1.29 | 1.17, 1.41 |  |
| eGFR (mL/min per 1.73 m^2^)* |  |  | <0.001 | … | … | … |
| 40 vs. 75 mL/min per 1.73 m^2^ | 1.61 | 1.26, 2.07 |  |  |  |  |
| 90 vs. 75 mL/min per 1.73 m^2^ | 1.24 | 1.04, 1.49 |  |  |  |  |
| CESD ≥8 | … | … | … | 1.47 | 1.23, 1.77 | <0.001 |
| Diabetes | … | … | … | 1.30 | 1.06, 1.60 | 0.012 |
| In the model for male participants, the effect of BMI and eGFR on not maintaining disability-free survival outcome were significantly nonlinear. In the model for female participants, the effect of BMI and Gait speed on the outcome were significantly nonlinear. All non-linear relations were modelled with a restricted cubic spline function with 2 degrees of freedom. To simplify interpretation of the corresponding regression coefficients, only HRs for 2 derived contrasts for each predictor are given. P values for those predictors are based on overall Wald tests of the restricted cubic spline function. Abbreviations: HR = Hazard Ratio, CI = Confidence Interval | | | | | | |

﻿

Table 3: Sex-specific formulas for calculation of the risk prediction model for disability-free survival within 5 years.

| **Model for Women:**  5-year risk (%) = (1 – (4.787198 x 10^-8^ )^exp (LP)^ ) x 100%, with:  LP = 0.0812863 x Age - 0.0785434 x 3MS - 2.1058784 x Gait speed + 13.6499575 x (Gait speed - 0.8391608)^3 *(If Gait speed > 0.8391608)* - 27.8419999 x (Gait speed - 0.9852217)^3 (*If Gait speed > 0.9852217*) + 14.1920424 x (Gait speed - 1.1257036)^3 (*If Gait Speed > 1.1257036*) - 0.0824954*BMI + 0.0022333 x (BMI - 24.48889, 0)^3 (*If BMI > - 24.48889*) - 0.0040281 x (BMI - 27.46667)^3 (*If BMI > 27.46667*) + 0.0017948 x (BMI - 31.17188, 0)^3 (*If BMI > 31.17188*) + 0.3885922*(CESD > 8) - 0.0188137 x Grip strength + 0.2644989 x Diabetes |
| --- |
| **Model for Men:**  5-year risk (%) = (1 – (5.304956 x 10^-8^)^exp (LP)^) x 100%, with:  LP = 0.0791378 x Age - 0.0614952 x 3MS - 0.9872901 x Gait speed - 0.0175855 x Grip strength - 0.6700744 x (Smoking = Former/Never) - 0.0809386 x BMI + 0.0034296 x (BMI - 25.29407)^3 *(If BMI > 25.29407)* - 0.0064518 x (BMI - 27.55675)^3 *(If BMI > 27.55675)* + 0.0030222 x (BMI - 30.12438)^3 *(If BMI > 30.12438)* - 0.0157298 x eGFR + 0.0000532 x (eGFR - 63.96903)^3 *(If eGFR > 63.96903)* - 0.0001092 x (eGFR - 74.24563)^3 (*If eGFR > 74.24563)* + 0.0000560 x (eGFR - 84.00224)^3 *(If eGFR > 84.00224)* |

Figure 1: Discrimination of each selected predictor, of their combination when added sequentially in order of their AUC, and of the final models.

| Women | Men |
| --- | --- |
| 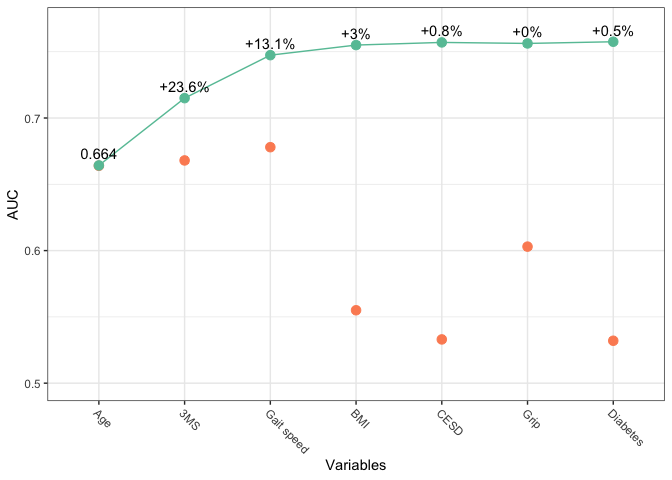 | 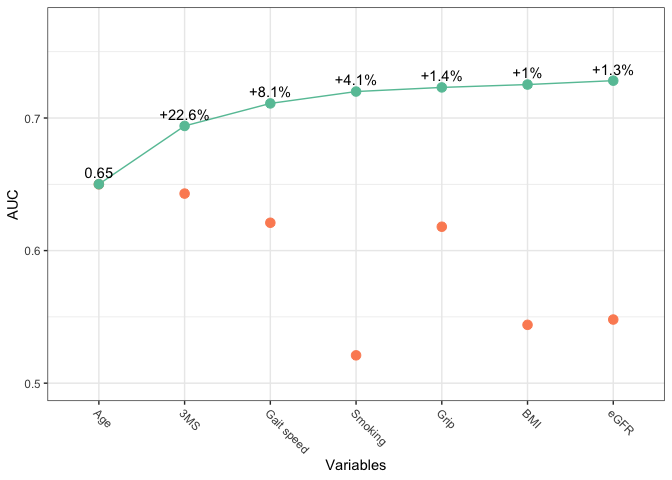 |

Orange dots show the AUC at 5 years of models made with each predictor of not maintaining disability-free survival individually. Green dots show the AUC of models made by incrementally adding each predictor along the x-axis. The percentages show the added value of the current model against the previous model. Predictors are arranged by their inclusion frequency. Abbreviation: AUC = Area under the curve

Figure 2: Calibration graph comparing the observed risk (based on Kaplan-Meier estimates) and the predicted risk with the final models, by tenths of the predicted probability. The red asterisk represents the bias-corrected predicted risks.

| 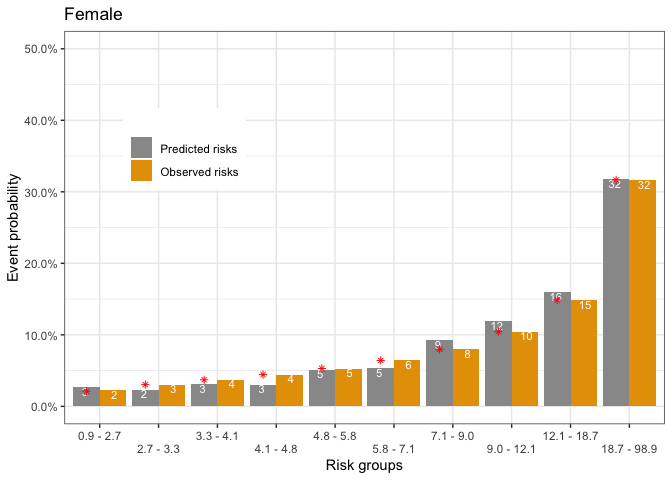 | 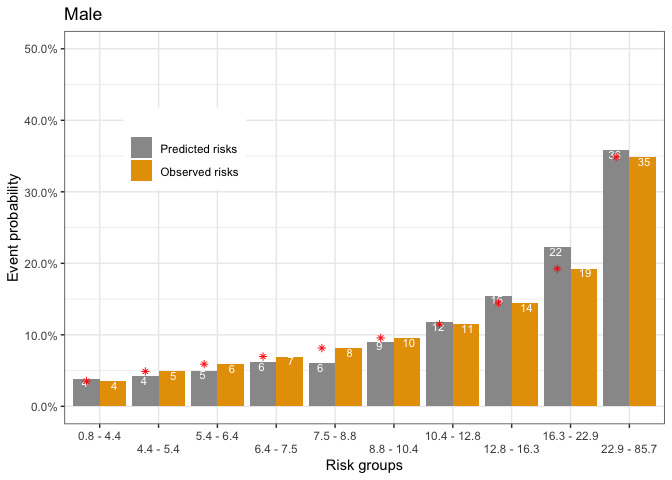 |
| --- | --- |
